# Monitoring and Determining Mitochondrial Network Parameters in Live Lung Cancer Cells

**DOI:** 10.3390/jcm8101723

**Published:** 2019-10-18

**Authors:** Tamara Mirzapoiazova, Haiqing Li, Anusha Nathan, Saumya Srivstava, Mohd W. Nasser, Frances Lennon, Brian Armstrong, Isa Mambetsariev, Peiguo G. Chu, Srisairam Achuthan, Surinder K. Batra, Prakash Kulkarni, Ravi Salgia

**Affiliations:** 1 Department of Medical Oncology and Therapeutics Research, City of Hope National Medical Center, Duarte, CA 91010, USA; tmirzapoiazova@coh.org (T.M.); anathan@coh.org (A.N.); ssrivastava@coh.org (S.S.); Imambetsariev@coh.org (I.M.); pkulkarni@coh.org (P.K.); 2 Center for Informatics, City of Hope National Medical Center, Duarte, CA 91010, USA; hali@coh.org (H.L.); sachuthan@coh.org (S.A.); 3 Department of Computational & Quantitative Medicine, Beckman Research Institute, City of Hope Medical Center, Duarte, CA 91010, USA; 4 University of Nebraska, Medical Center, Nebraska, NE 68198, USA; wasim.nasser@unmc.edu (M.W.N.); sbatra@unmc.edu (S.K.B.); 5 Abbott Molecular, Des Plaines, IL 60018, USA; franlen@gmail.com; 6 Department of Developmental and Stem Cell Biology, City of Hope National Medical Center, Duarte, CA 91010, USA; BArmstrong@coh.org; 7 Department of Anatomic Pathology, City of Hope National Medical Center, Duarte, CA 91010, USA; PChu@coh.org

**Keywords:** mitochondria, dynamics, morphology, imaging, fractals, lung cancer, scanning electron microscopy

## Abstract

Mitochondria are dynamic organelles that constantly fuse and divide, forming dynamic tubular networks. Abnormalities in mitochondrial dynamics and morphology are linked to diverse pathological states, including cancer. Thus, alterations in mitochondrial parameters could indicate early events of disease manifestation or progression. However, finding reliable and quantitative tools for monitoring mitochondria and determining the network parameters, particularly in live cells, has proven challenging. Here, we present a 2D confocal imaging-based approach that combines automatic mitochondrial morphology and dynamics analysis with fractal analysis in live small cell lung cancer (SCLC) cells. We chose SCLC cells as a test case since they typically have very little cytoplasm, but an abundance of smaller mitochondria compared to many of the commonly used cell types. The 2D confocal images provide a robust approach to quantitatively measure mitochondrial dynamics and morphology in live cells. Furthermore, we performed 3D reconstruction of electron microscopic images and show that the 3D reconstruction of the electron microscopic images complements this approach to yield better resolution. The data also suggest that the parameters of mitochondrial dynamics and fractal dimensions are sensitive indicators of cellular response to subtle perturbations, and hence, may serve as potential markers of drug response in lung cancer.

## 1. Introduction

Mitochondria are highly dynamic organelles whose number and morphology within a given cell vary with origin, differentiation stage, energy requirements, and stage of the cell cycle [1,2]. However, variation appears to be regulated in order to maintain energy and metabolic homeostasis while deregulation of mitochondrial dynamics is associated with the onset of metabolic dysfunction and disease [3]. Thus, many aspects of mitochondrial dynamics, morphology, regulation, and intracellular organization are found to be cell type and tissue specific [4]. Alterations in mitochondrial biogenesis, and degradation have been widely linked to pathological states, such as cancer, aging, and degenerative diseases [5,6,7,8]. Furthermore, mechanistic studies of mitochondrial dynamics have shown that mitochondrial division (due to fission) and elongation (due to fusion) are mediated by key proteins, including dynamin-1-like protein (DNM1L), also known as dynamin related protein 1 (DRP1), a mechano-chemical GTPase, and mitofusin 1 (MFN1) and mitofusin 2 (MFN2), respectively [9,10,11]. The dynamic nature of the mitochondria, and the potential mechanistic connections between their morphology and cell state, tends to suggest that the salient features of these subcellular organelles could have tremendous biomedical potential. 

For example, the parameters used to discern mitochondrial dynamics could serve as valuable markers for disease diagnosis, prognosis, and treatment. Despite this potential utility however, finding reliable and quantitative tools for monitoring mitochondria in live cells has proven challenging, and only a few attempts have been reported in the literature [12,13]. For example, recently, Harwig et al. published the MitoGraph method for analysis of mitochondrial morphology in live and fixed cells, with general recommendations for confocal imaging of labeled mitochondria [14]. This study showed that MitoGraph may also be useful for image analysis of intravital microscopy of live animals [15,16]. Mitochondria-based cancer treatment can be developed by imaging mitochondria in living cells. Additional studies investigating image-based approaches are required to understand the mechanisms by which mitochondria regulate cell fate [17,18].

In this manuscript, using high resolution confocal fluorescence microscopic images of live cells, we compared the mitochondrial network of SCLC cells and non-malignant BEAS-2B cells. Based on the balance between fusion and fission balances of the mitochondrial network—the two opposite processes that regulate mitochondrial number, size, and positioning within the cytoplasm [19]—we describe a quantitative imaging-based approach that combines automatic mitochondrial morphology and dynamics analysis with fractal analysis. We refer to this approach as ‘MitoMorF’. MitoMorF serves dual functions. First, it quantifies mitochondrial dynamics, which we define as the speed, length, and wiggle ratio. Second, because mitochondrial networks display self-similar properties of fractals, it uses fractal analysis to determine the complexity of these networks within cells. This image-based assay can be used for profiling high-quality images of mitochondria to characterize mitochondrial dynamics and network connectivity in living cells. Measurements of these parameters will help improve the knowledge of mitochondrial dynamics and serve as biomarkers for early detection of SCLC and indications of the aggressiveness of tumors. 

## 2. Experimental Section

### 2.1. Tissue Microarray

Small cell lung cancer tissue microarrays (TMAs) were from US Biomax Inc. (Rockville, MD, USA; LC818). Immunohistochemical (IHC) staining was performed using standard techniques previously described [20] with antibodies against Dynamin 1 like protein (DRP1) (EMD Millipore, Billerica, MA, USA), B-cell lymphoma 2 (Bcl-2) (ThermoFisher, Scientific, Carlsbad, CA, USA), and Mitofusion 2 (Mfn2) (ThermoFisher, Scientific, Carlsbad, CA, USA) in the pathology/solid tumor core of The City of Hope National Medical Center. Briefly, each TMA was reviewed and scored by two independent pathologists on a scale of 0 to 3: 0+, no staining, no expression; 1+, weak staining, low expression; 2+, moderate staining, moderate expression; and 3+, strong staining, high expression. Regression analysis was performed in Python using scipy (https://www.scipy.org/).

### 2.2. Cell Culture Reagents

BEAS-2B, H82, H526 cells were purchased from ATCC (Manassas, VA, USA) and DMS273 adherent SCLC cells were gifted from Dr. Yusuke Nakamura (University of Chicago). Cell culture media RPMI 1640, phosphate buffer saline (PBS), L-glutamine, penicillin-streptomycin, sodium bicarbonate, and HEPES were purchased from Thermo Fisher Scientific (Waltham, MA, USA). Fetal bovine serum (FBS) was from Sigma (St. Louis, MO, USA). 

### 2.3. Preparation of BEAS-2B and SCLC Cells for Scanning Electron Microscopy

Sample blocks were prepared for serial block-face scanning electron microscopy (SBF-SEM) as described [12] with minor modification. Cells were fixed in 0.15 M cacodylate buffer (pH 7.4) containing 2.5% glutaraldehyde with 2 mM calcium chloride. After primary aldehyde fixation, the colonies were rinsed in 0.15 M sodium cacodylate containing 2 mM calcium chloride and post fixed in 1.5% potassium ferrocyanide-reduced 2% osmium tetroxide in 0.15 M cacodylate buffer for 1 h. Tissue was then rinsed in distilled water and treated with 0.1% aqueous thiocarbohydrazide for 20 min. After further rinsing in distilled water, the tissue was again treated with 2% osmium tetroxide for 30 min, rinsed in distilled water, dehydrated in an ethanol series, and infiltrated with Durcupan ACM resin. 

### 2.4. Serial Block-Face Scanning Electron Microscopy (SEM)

The sample blocks were mounted on an aluminum pin and trimmed to 0.5 mm × 0.5 mm in size. The specimen was placed in a field-emission scanning electron microscope (Zeiss Sigma VP) equipped with a serial block-face sectioning unit (Gatan 3View) and a backscatter electron image of the face obtained under an accelerating voltage of 4 KV and a chamber pressure of 20 Pa (variable pressure mode). An automatic microtome then removed a 70-nm thick slice from the face and another image was recorded. This procedure was repeated 1000 times to give a data set from which a complete three-dimensional reconstruction could be derived.

### 2.5. Mitochondrial Staining in Live Cells and Confocal Microscopic Imaging

To observe the mitochondrial network in BEAS-2B and DMS273 cells, cells were seeded at 20,000/35-mm glass bottom dish (MatTek) and allowed to adhere overnight. Cells were stained the next day for 60 min at 37 °C in a 5% CO_2_ incubator with 20 nM MitoTracker Deep Red FM /700 nM Hoechst (Life Technologies, Carlsbad, CA, USA). For mitochondrial division inhibitor 1 (MDIVI-1) treatment, cells were washed once in phosphate buffered saline (PBS) and treated with MDIVI-1 in 1% serum media for 60 min. Cells were washed three times with phenol-red free RPMI (corning) and then were incubated in live cell imaging solution (Life Technologies). Then, 35-mm coverslip bottom dishes were placed on the microscope stage inside a heated chamber at 37 °C and 5% CO_2_. Time-lapse images were acquired using an inverted Carl Zeiss LSM880 utilizing the Airyscan Detector and an LCI-PlanNeofluar 63 × /1.3 Immersion objective and Immersol (Carl Zeiss). Image scaling was done at 0.04 µm per pixel in relation to 3x zoom. Images were acquired using optimal Airyscan parameters and Airyscan images were post-processed using the Zeiss Zen Black Airyscan processing function. 

### 2.6. Integrating Imaging 

The integrated workflow embodied in MitoMorF entailed capturing live cell mitochondrial videos using high resolution confocal laser scanning microscopy LSM880 (Zeiss). The video was recorded for, on average, one frame every 12 seconds in order to capture super high-resolution images. A total of 24 frames per cell were recorded. Mitochondrial branch detection and motion analysis were done using Mytoe [21]. Mitochondrial network images were segmented using Otsu thresholding with a white top-hat filter and median filter. In addition to the Mytoe default filters, each frame was preprocessed using Fiji/ImageJ’s Gaussian blur filter with a kernel size of three to further remove background noise in the images. Neighborhood analysis of these segmentation masks was used to determine pixel connectivity. The eight surrounding pixels (imagine a 3 x 3 grid with the pixel of interest being the centermost square) were processed to identify the connection status. For example, 8-connected pixels are connected horizontally, vertically, and diagonally, i.e., they are connected to every pixel that shares an edge or corner. Connected pixels were given a value of one, and pixels that were not connected were given a value of zero. Pixels were denoted as branch points if they had at least three neighborhood pixels with a value of one, and pixels were denoted as branch endpoints if there were at least four neighborhood pixels with a value of zero [21]. Branch points represent crossing of the mitochondrial network while endpoints (branch terminals) were used to calculate branch size (length). 

Mitochondrial branch structures were generated as a mask file for mitochondrial size measurements. The motion of mitochondrial branches between each frame of the live cell video was computed by optical flow estimation using a modified Pyramidal Lucas–Kanade (PLK) implementation [22] shown in Equation (1) [21]:

(1) $$\left[\begin{array}{c} u\\ v \end{array}\right]=\;\begin{array}{c} {\left[\begin{array}{c} G*\left(I_{x}{}^{2}\right)\;G*\left(I_{x}I_{y}\right)\\ G*\left(I_{x}I_{y}\right)\;G*\left(I_{y}{}^{2}\right) \end{array}\right]}^{-1} \end{array}\left[\begin{array}{c} -G*\left(I_{x}I_{t}\right)\\ -G*\left(I_{y}I_{t}\right)\; \end{array}\right],$$

where *u* and *v* are the displacement of the image, *I*, along the on x and y coordinate axes, *G* is a Gaussian kernel, *I_x_* and *I_y_* are the spatial derivatives, and *I_t_* is the time derivative. A detailed description of the algorithm can be found in [21]. The optical flow estimation computes the displacement (*u*, *v*) of each pixel on the branches. The branch speed is the average of the speed of each pixel in the branch, shown in Equation (2) [21]:

(2) $$\bar{S}=\sum_{i=1}^{N}||V_{i}||,$$

where *V* is velocity vector of each pixel on the branch. Besides the speed, the optical flow estimation provides detailed measurement to compute the directedness moving pattern wiggle ratio, which is defined as the ratio of the mean of absolute vectors over the absolute value of the mean vector [23], shown in Equation (3) [21]:

(3) $$W=\frac{\bar{S}}{\left||\bar{V}|\right|},$$

where *s* is the speed and *v* is the velocity vector of each pixel on the branch. The mitochondrial branch mask of the first frame generated was used for fractal and multifractal analysis. Fiji/ImageJ’s Fraclac plugin [24] was used to calculate the fractal dimension (FD), lacunarity, and singularity spectrum. The program is freely accessible online. Fractal analysis and multifractal analysis was established using the standard box counting scan method. 

### 2.7. Mono-Fractal Analysis

Mono-fractal analysis measures the complexity and heterogeneity within an image. It generates two measurements: Fractal dimension (FD) and (Λ) In this study, fractal analysis was performed on the mitochondrial network mask of the first frame of the mitochondrial video using the box counting scan method implemented in the FracLac plugin version 2015Sep090313a9330 from ImageJ. The box counting scan method covers the image with a grid of boxes at various scales. As the image is zoomed in on, the box size becomes smaller and smaller. It then determines the number of boxes necessary to cover the object (foreground pixels) at each scale. FracLac estimates the FD as the slope of the regression line for the log-log plot of box size/scale and count. It is defined in Equation (4):

(4) $$FD=\;-\underset{\varepsilon \to 0}{\lim}\frac{\ln N\varepsilon }{\ln\varepsilon },$$

where Nε is the number of boxes needed to cover the object in the image at a specific scale, ε [FracLac Manual]. 

Lacunarity is a measure of the heterogeneity in an image. FracLac estimates the lacunarity by the object (foreground pixel) mass distribution per box, defined in Equation (5):

(5) $$\Lambda_{\varepsilon }=\;{\left(\frac{\sigma }{\mu }\right)}^{2},$$

where σ is the standard deviation of the object pixels per box at scale ε and *µ* is the mean of the object pixels per box at scale ε. In this study, we reported the average lacunarity ($\Lambda_{av}$), which is the mean of the lacunarities at all box sizes/scales:

(6) $$\Lambda_{av}=\;\frac{\sum \Lambda_{\varepsilon }}{E},$$

where *E* is the total number of box scales. 

### 2.8. Multifractal Analysis

Multifractal analysis is used to describe data that exhibit a non-linear power-law behavior. Essentially, it describes signal regularity of scale-free phenomena. This kind of analysis characterizes scaling behavior with respect to various statistical moments. Mono-fractal datasets require only a single scaling exponent or a linear combination of the exponents to be characterized whereas multifractal datasets require nonlinear functions of the datasets to be characterized. In multifractal analysis, we usually use a spectrum diagram in order to distinguish the multifractal, mono-fractal, and non-fractal images. In this study, we use D_Q_ vs. Q spectra diagrams, where D_Q_ is the generalized dimension and Q is an arbitrary set of exponents. If the dataset has multifractal status, the D_Q_ vs. Q spectra is a sigmoidal curve. If the image has mono-fractal status, the D_Q_ vs. Q spectra is a linear as Q increases. For non-fractal images, the D_Q_ vs. Q spectra is a horizontal line. 

Here, multifractal analysis was performed using the distribution of pixel values (mass distribution) through the box counting scan method implemented in the FracLac plugin version 2015Sep090313a9330 from ImageJ. We report generalized fractal dimensions $\left(D_{\left(Q\right)}\right)$ and two multifractal spectra: The generalized dimension spectrum $\left(D_{\left(Q\right)}\;vs.Q\right)$ and the singularity spectrum $\left(f_{\left(\alpha \right)\;}vs.\;\alpha \right)$. 

Generalized fractal dimension, $D_{\left(Q\right)}$, measures how mass varies with scale, ε, in an image. It provides the global measurement of the whole mitochondrial network. It is calculated as follows:

(7) $$Q\neq 1,\;\;D_{\left(Q\right)}=\;\frac{\underset{\varepsilon \to 0}{-\lim}\frac{\ln I_{\left[Q,\varepsilon \right]}}{\ln\varepsilon }}{1-Q},$$

where Q is an arbitrary exponent and $I_{\left[Q,\varepsilon \right]}$ is the moment of Q, which is defined as follows:

(8) $$I_{\left[Q,\varepsilon \right]}=\;\sum_{i=1}^{N}{\left[P_{\left(i,\varepsilon \right)}\right]}^{Q},$$

where $P_{\left(i,\varepsilon \right)}$ is the probability distribution of the mass for all boxes at scale, ε. Multifractal analysis was performed on the same mitochondrial mask images using FracLac plugin version 2015Sep090313a9330 from ImageJ with a range of Q = −10 to 10.9 with increments of 0.1.

We report three popular generalized fractal dimensions: Capacity dimension ($D_{\left(0\right)})$, information dimension ($D_{\left(1\right)}$), and correlation dimension ($D_{\left(2\right)}$). Capacity dimension ($D_{\left(0\right)})$ is the same as the box counting dimension (FD) in monofractal analysis, which is defined by the relationship between the number of boxes that cover the object in an image at various scales, ε.

Information dimension ($D_{\left(1\right)}$) is related to information theory, and measures how the average information needed to identify an occupied box changes as the scale gets smaller. The algorithm for the information dimension searches for the linear relationship between the logarithm of a box size (ε) and the logarithm of the probability, P, that a given box contains an element of the object in the image. In image analysis, the information dimension captures the evenness of the data intensities in space. At Q = 1, $D_{\left(Q\right)}$ is defined as:

(9) $$Q=1,\;\;D_{\left(Q\right)}=\;\underset{\varepsilon \to 0}{-\lim}\frac{\sum_{i=1}^{N}\left[P_{\left(i,\varepsilon \right)}*\ln P_{\left(i,\varepsilon \right)}\right]}{\ln\varepsilon }.$$

The correlation dimension ($D_{\left(2\right)}$) is a probability measure of two pixels within the object being within ε of each other. It calculates not only whether a given box is occupied by a pixel of the analyzed geometric object but also how many pixels there are in the box and how close to each other they are. At Q = 2, $\;D_{\left(Q\right)}$ is defined as:

(10) $$Q=2,\;\;D_{\left(Q\right)}=\;\underset{\varepsilon \to 0,M\to \infty }{-\lim}\frac{\ln(\frac{g_{\varepsilon }}{M^{2}})}{\ln\varepsilon },$$

where *M* is the number of pixels of the analyzed object in the image, and $g_{\varepsilon }$ is the number of pairs of pixels that are within ε of each other [25]. In general, $D_{\left(Q\right)}$ of an object with multifractal properties is a decreasing function, where $\;D_{\left(0\right)}$ ≥ $D_{\left(1\right)}$ ≥ $D_{\left(2\right)}$.

Beside these three generalized dimensions, we also report the generalized dimension spectrum $\;(D_{\left(Q\right)}\;vs.Q)$ and the singularity spectrum $\left(f_{\left(\alpha \right)\;}vs.\;\alpha \right)$. These multifratal spectrums provide details of the dimensions at various scales. The singularity spectrum $\left(f_{\left(\alpha \right)\;}vs.\;\alpha \right)$ represents the multifractal property using a non-integer exponent, α, known as the Hölder exponent, which describes the local singularity of the object in the image. With known $\;D_{\left(Q\right)}$, the singularity spectrum is defined as:

(11) $$\alpha \left(Q\right)=\frac{D_{\tau \left(Q\right)}}{D_{Q}}f\left(\alpha \left(Q\right)\right)=Q\alpha \left(Q\right)-\tau \left(Q\right),$$

where α is the singularity strength and $\tau \left(Q\right)=\left(Q-1\right)\times D_{Q}$ [26].

### 2.9. Statistical Analysis

The Wilcoxon rank-sum test was performed to test if two independent samples were selected from populations with the same distribution. The *p*-value indicates the level of distance between the two data sets. All tests were performed in Python using scipy (https://www.scipy.org/) and numpy (http://www.numpy.org/) and were visualized using seaborn (https://seaborn.pydata.org).

### 2.10. 3D Reconstruction of SCLC Mitochondria

3D SCLC mitochondria network were reconstructed from the 3D electron microscopy images of mitochondria. The mitochondria regions were segmented using the segmentation editor from the commercial software AMIRA version 6.5.0 (Thermo Fisher Scientific). Interactive image segmentation was used to achieve highly accurate result. The segmentation labels were used to generate the 3D volume of the mitochondrial network, which was used for downstream 3D fractal dimension analysis. 

## 3. Results

### 3.1. The Mitochondrial Fission-Associated Protein DRP1 Expression is Increased in SCLC

**Figure 1:**
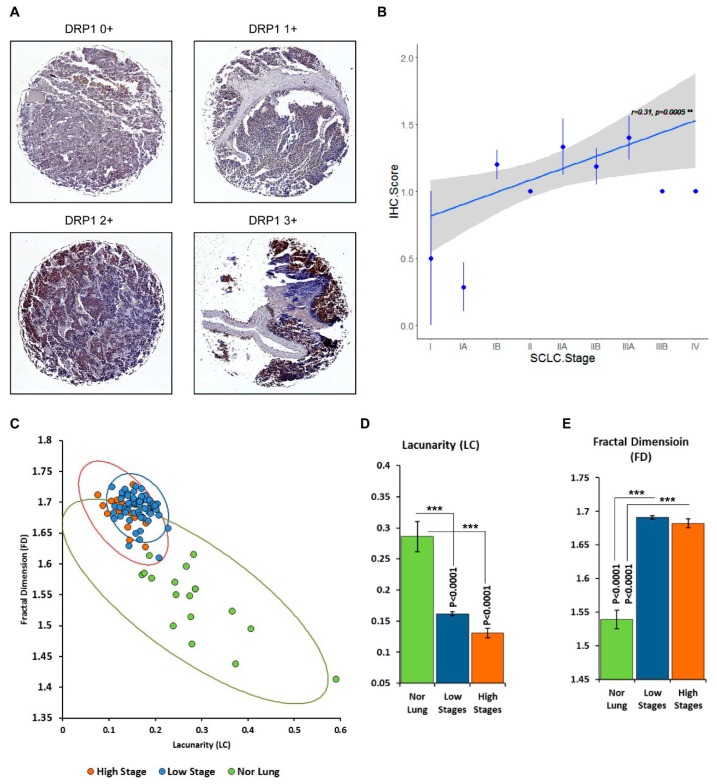
Upregulation of dynamin 1 like protein (DRP1) in human small cell carcinomas. (**A**) Representative images of immunohistochemical staining of DRP1 in tissue microarray (TMA) sections. Detailed procedures are described in the materials and methods. DRP1 immunostaining was moderated (1+ /2+) in 69 tumors and score 3+ was observed in one tumor stage III disease. (**B**) Correlation analysis confirmed a significant positive correlation between the DRP1 score and stage of tumor (*r* (78) = 0.31, *p* = 0.005) using R (v3.2.3) for quantitate analysis. The blue dots are the average IHC score at each cancer stage and the blue bar is the standard error. The blue line is the regression line and the gray area is the 95% confidence interval. The diagram was generated using R’s ggplot2 library. (**C**) Normal lung tissue and small-cell carcinoma were scanned and the images were then converted to 8-bit greyscale using ImageJ**^25^**. The box-counting FD (*D*_B_) and lacunarity (Λ) of specimens of normal lung tissue (*n* = 18) and histological variants of SCLC tumors (*n* = 79) were calculated using FracLac [18]. Tumors were separated to low-stage (*n* = 65) and high-stage groups (*n* = 14). The low-stage group includes stage I, II, IIA, and IIB samples. The high-stage group includes IIIA, IIIB, and IV samples. Scatter plot of lacunarity vs. fractal dimension with confidence ellipses per tissue type (95% confidence interval). (**D** and **E**) Graphical representation of mean values of LC and FD in TMA sections of lung normal and tumor tissues. Error bars = standard error. *p-*values indicate significance from one-way variance analysis (ANOVA). *** *p* < 0.0001.

We first determined the expression of the key molecules associated with mitochondrial fission and fusion namely, DRP1, MNF2, and BCL2. We performed immunohistochemistry using SCLC tissue microarrays (TMAs) (US Biomax, Rockville, MD). TMA staining was quantified as ranging from 0 to 3 using a semi-quantitative visual index described in the methods section. Representative images of immunohistochemical staining of DRP1 are shown in Figure 1. In total, 80 cases were screened (Supplementary Table S1) and only DRP1 immunostaining was observed to increase in intensity with disease progression (Figure. 1A). Correlation analysis confirmed a significant positive correlation between the DRP1 score and stage of tumor (*p* = 0.005, *r* (78) = 0.31) (Figure 1B). In contrast DRP1, both MFN2 and BCL2, did not show any significant differential expression (Supplementary Figure S1). Of note, since IHC grading could be biased and affected by the individual observer, as well as the fact that this analysis is deemed highly qualitative, we would like to emphasize that the analysis presented in Figure 1B was used to infer a correlation between the tumor stage and expression of mitochondrial fission protein DRP1.

The irregularity of tumor growth leads to abnormal images of lung cancer tumors. Classical Euclidean geometry measurement of volume, density, and size can distinguish only visible configurations of the tumor. However, information like texture and statistical properties of shape that are hidden in structural complexity can often not be characterized using conventional Euclidean geometry. Our previous studies have shown that tumor structures have fractal properties [27]. Fractals are infinitely complex patterns that are self-similar across different scales [28]. Objects that exhibit exact, quasi, or statistical self-similarity may be considered fractal, and we have found that mitochondrial networks within cells display fractal properties. 

A fractal dimension measures the complexity of an image as it is scaled, while lacunarity is a numerical description of the texture of an image and is thus considered a measure of the image’s heterogeneity [29]. Therefore, measurements of fractal dimension (FD) together with lacunarity (LC) can be used to indicate the complexity and space-filling properties of a shape [30]. Thus, we used FD and LC analysis to differentiate between normal tissue, adjacent tissue, and malignant SCLC lung tissue. We analyzed SCLC and normal lung TMA slides stained with DRP1 for FD and LC measurements. Both parameters have significant differences between normal lung tissue and small cell carcinoma. While, the mean value of normal tissue FD was 1.5392 ± 0.0142 and LC was 0.2861 ± 0.0245, all tumor tissues had higher values of FD and lower values of LC (Figure 1C–E, and Supplementary Table S2). However, FD measurements were unable to discriminate between the various disease stages at statistically significant levels, most likely due to the small number of samples in many cases. On the other hand, LC values were able to differentiate between early (I-IIB)- and late (IIIA-IV)-stage tumors (Figure 1C). LC values in early-stage tumors were higher compared to late-stage tumors, which is consistent with our previous finding that LC is considerably decreased in lung cancer subtypes compared with normal lungs [31]. 

### 3.2. Live Cell Imaging to Monitor Mitochondrial Morphology and Dynamics Using MitoMorF

Next, we elucidated the changes in shape, moving pattern, and speed of intracellular movement of the individual mitochondria in SCLC cells. Several imaging-based attempts have been made in the past to address mitochondrial dynamics by segmenting mitochondria from fluorescence microscope images [32,33,34,35]. However, with very few exceptions [13], imaging is performed on fixed cells and the tools are semi-quantitative and rely on human interaction. Furthermore, to the best of our knowledge, mitochondrial FD have not been evaluated in conjunction. 

In our experiments, we used BEAS-2B and DMS27 cell lines. The former represents a non-tumorigenic cell line while the latter a tumorigenic one. They are both derived from the epithelial lining of the lung, and, therefore, represent models to study ‘normal’ and ‘cancer’ lung tissue. Furthermore, given their common lineage, any differences observed in the mitochondria may be attributable to the effect of pathology. 

**Figure 2:**
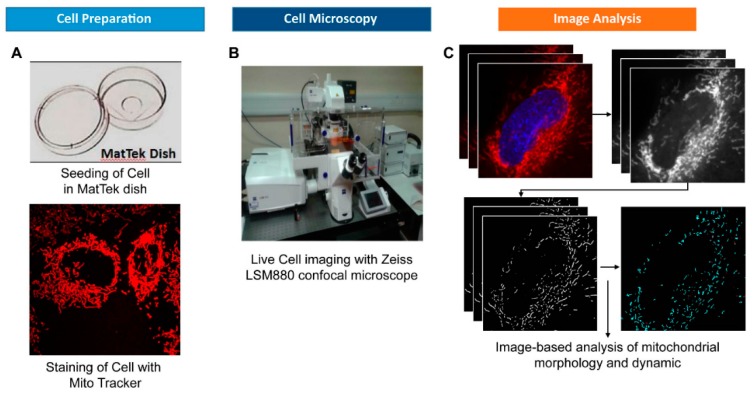
Workflow of the quantitative analysis of mitochondrial networks in live cells. (**A**) Basic instruction for mitochondrial network staining with Mito Tracker Red and cell imaging. (**B**) The living cell mitochondrial video was captured using high resolution confocal laser scanning microscopy LSM880. The video was recorded at an average of 12 seconds per frame in order to capture super high-resolution images. A total of 24 frames of video per cell were recorded for mitochondrial dynamic analysis. (**C**) Each frame was preprocessed using Fiji/ImageJ’s Gaussian blur filter with kernel size 3 to remove the background noise. The filtered images were analyzed using Mytoe to detect the detail of the mitochondrial branch, and to measure its size, moving speed, and moving patterns.

MitoMorF integrates FracLac [24] with Mytoe [21], a segmentation method that identifies individual branches of the organelle’s structure by thresholding and morphological image processing. Mytoe detects each individual branch by using the branch point and end point analysis based on the connectivity status of eightt neighborhoods of each point on the branch. Mytoe allows quantification of both single branches and the macroscopic structure formed by the mitochondria. It implements the pyramidal Lucas–Kanade algorithm [22] using MATLAB for the optical flow motion estimation and analysis [36]. Optical flow estimation is a well-established method for automated tracking and motion measurement. It has been shown that the optical flow method is capable of detecting and measuring the fast movement of mitochondrion [37] and can provide unbiased measurement in a large range of speeds for mitochondrial movement. Meanwhile, optical flow estimation computes the displacement of each pixel on the branch. Beside the average speed of the branches, this method also provides detailed measurements to compute the wiggling motion pattern of mitochondrial branches. Thus, the output was examined in Mytoe with branch-level data being visualized as color-coded individual branches and motion vectors as quiver plots. We used the mitochondrial mask of the first frame of the living cell movie from the Mytoe result for the fractal analysis. A flow diagram integrating the fractal analysis with Mytoe is shown in Figure 2 and the scanning parameters used to acquire high-resolution images are shown in Supplementary Table S3. 

**Figure 3:**
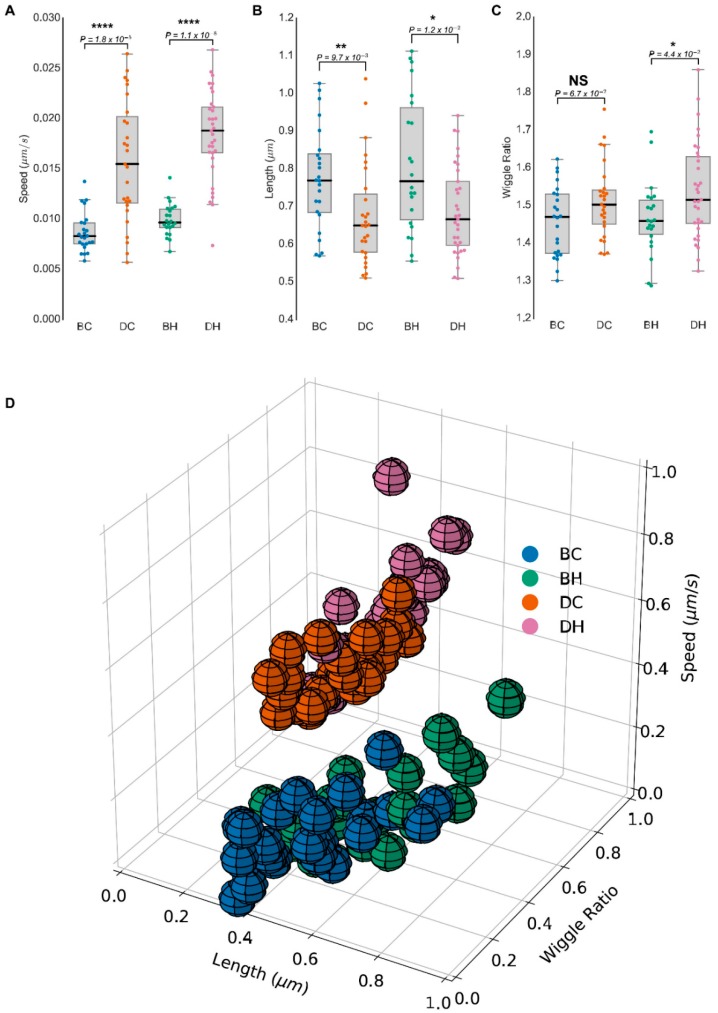
Mitochondrial network activity in live BEAS-2B and SCLC cells. (**A**) The living cell mitochondrial video was captured using high resolution confocal laser scanning microscopy LSM880 and Airyscan-processed images were analyzed using Mytoe to measure speed. Box plots of the average cell speed present a significantly higher speed in the SCLC cell line DMS273 before and after stimulation with HGF (100 ng/mL). (**B**) Box plots present the average cell length. Cell length was defined as the median number of each cell’s mitochondrial network frame. (**C**) Box plots present the wiggle ratio in four cell groups BC–DC and BH–DH. * *p* < 0.05; ** *p* < 0.01; **** *p* < 0.00001 values indicate significance from the Mann–Whitney test in all three panels. (**D**) The 3D scatter plot shows the relationship between the length (X-axis), wriggle ratio (Y-axis), and speed (Z-axis). (**E**) Images of the mitochondrial network before and after segmentation.

Employing MitoMorF, we compared the mitochondrial morphology and dynamics in the DMS273 SCLC cell line and the non-tumorigenic BEAS-2B lung cell line as a control. A total of 106 cells in 4 groups, namely BEAS-2B control (BC), BEAS-2B treated with HGF (BH), SCLC control (DC), and SCLC treated with HGF (DH), were imaged. We measured three mitochondrial parameters namely, length, speed, and wiggle ratio of the mitochondrial branches. The length and speed of the mitochondrial branch are two measurement parameters of the mitochondrion and are involved in controlling its fusion and/or fission cycles. The wiggle ratio is a measure of the directedness of mitochondrial movement [23]. It is the ratio between the mean of N measured points’ absolute velocities in a mitochondrial branch and the mean of their directional velocities. If the entire mitochondrion moves to the same direction, the wiggle ratio is close to 1. If different parts of the mitochondrion are moving in different directions, the mean of the absolute velocities will be greater than that of the directional velocities, and the wiggle ratio will be >1. The wiggle ratio can be used to describe the moving pattern of the mitochondrial branches. Thus, a large wiggle ratio means that each part of mitochondrial branch moves along different directions, but the whole branch shakes locally. The box plots show the difference between control cells and SCLC cells (Figure 3A–C). The Mann–Whitney test indicates that significant differences exist between normal cells and SCLC cells regarding these three parameters: (i) The moving speed of the SCLC cell’s mitochondrial branch is faster than that of the control cell (Figure 3A), (ii) the SCLC cell’s mitochondrial branch is significantly shorter than that of the normal cell (Figure 3B), and (iii) the wiggle ratio shows that the SCLC cell’s mitochondrial branch moves back and forth in the same location (Figure 3C).

Consistent with earlier reports demonstrating that mitochondria are more dynamic in cancer cells than their non-cancerous counterparts [8], we also observed that mitochondrial speed was higher in DC cells than that seen in the BC cells. Measurement of mitochondrial speed in DH cells after HGF stimulation was significantly higher compared with HGF-stimulated BH cells. However, we found a significant decrease in the average mitochondrial length in DC and DH groups compared with control BC and BH cells. In contrast, the wiggle ratio was higher in cancer cells compared with their non-cancerous counterparts. These mitochondrial morphometrical parameters representing an average of 23 frames of speed, length, and wiggle ratio are displayed as a 3D plot (Figure 3D), which suggest that fragmented mitochondria with high local movements, mostly wiggling, are seen in SCLC cells compared to control. Together, these results show that an integrated, imaged-based analytical approach of mitochondrial morphology and dynamics can distinguish non-malignant and SCLC cells. However, the image-based “MitoMorF” assay can be specifically used for profiling high-quality images of mitochondrial network dynamic and mitochondrial morphological measurement in living or fixed cells. However, measurement of mitochondrial speed, wiggle ratio, and multifractal analysis can only be done in living cells. 

### 3.3. Mono-Fractal and Multifractal Analysis

In addition to the speed, length, and wiggle ratio, we also assessed FD and LC analysis of the mitochondrial network. The details of the fractal analyses are described in the methods. FD indicates how much space is filled by the branches, and LC represents how the branches fill the space [38]. 

Depending on the cell type and the cell’s physiological status, mitochondria present as either small fragmented organelles or large interconnected networks. A high FD indicates highly clustered and complex mitochondrial morphology whereas a low FD is indicative of more open space. On the other hand, high lacunarity is indicative of a fractal with large holes and the converse is true for low lacunarity. In this way, high fractal dimensions are typically associated with low lacunarities. Thus, we used mono-fractal analysis within an image to measure mitochondrial networks’ complexities and heterogeneities.

**Figure 4:**
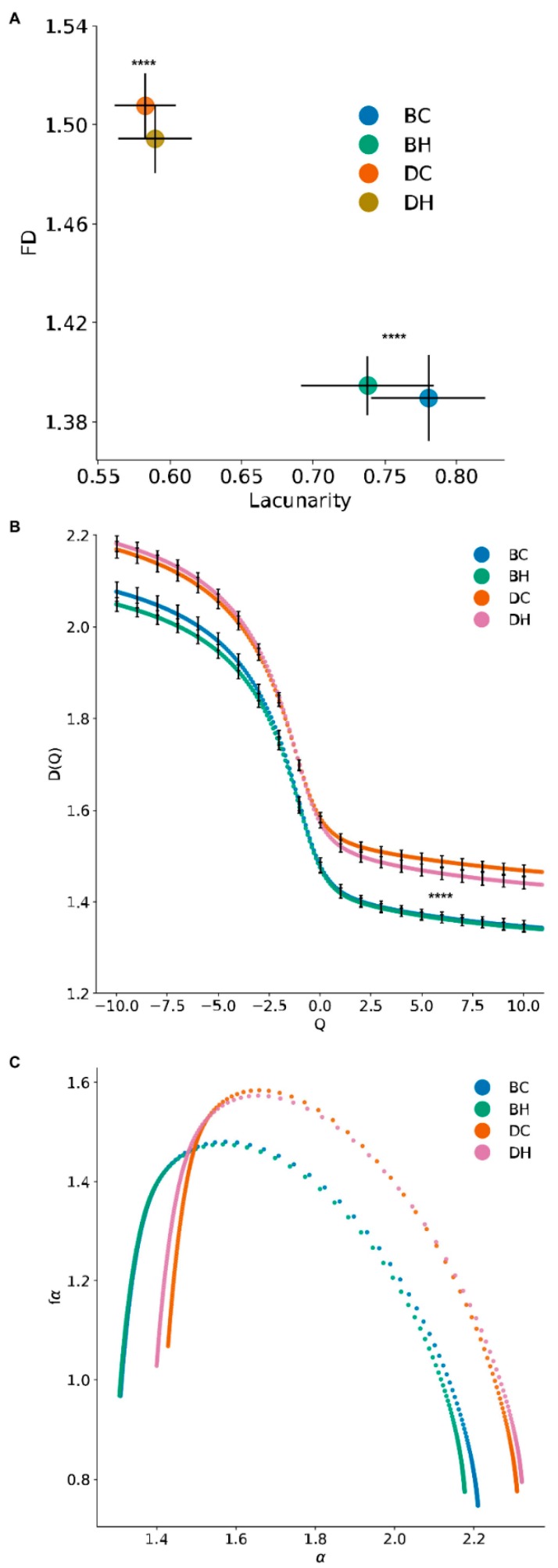
Fractal, lacunarity, and multifractal analysis of the mitochondrial network. (**A**) Mono-fractal and lacunarity analysis of the mitochondrial network. (**B**) Generalized dimensions of all mitochondrial regions for BC-BH and DC-DH. (**C**) Singularity spectrum f (α) versus α of BEAS-2B and DMS273 cells BC, BH, DC, and DH, respectively. **** *p* < 0.00001 values indicate significance from the Mann–Whitney test between control BC–BH cells and DC–DH tumor cells. Exact *p*-values for fractal dimension curves are reported in Table 1.

Previously, we showed that FD and LC take into account the fission/fusion states of mitochondrial networks [27]. Consistent with our earlier results, here we have shown that mitochondria are much more fragmented in the DC and DH cell groups compared to control BC and BH cells (Figure 3). Next, we applied the FD and LC metrics to the quantification of the mitochondrial network. A scatter plot of the mono-fractal analysis showed that SCLC DMS273 cells had high FD but a low LC index compared to BEAS-2B cells (Figure. 4A). Compared with the normal cells, the cancer cells have significant larger FD (*p < 0.00001*) and smaller lacunarity (*p < 0.00001*). These parameters correlated with the morphological profile of mitochondria in both cell types. Again, DMS273 cells had fragmented, dense mitochondria with limited elongation and BEAS-2B cells had a tubular and elongated shape. 

A multifractal analysis (generalized dimensions spectrum and singularity spectrum) further revealed that the images exhibit more than one fractal dimension due to inherent spatial irregularities [39]. Its results were presented in multifractal spectra that represent how a pattern behaves if amplified in certain ways [24]. The multifractal analyses indicated a significant difference in the generalized dimension’s spectrum curves between the non-cancerous and SCLC cells. The generalized dimensions spectrum (Figure 4B) shows that the SCLC cell’s generalized fractal dimensions are larger than those of normal cells at all scales, i.e., across all exponents (Q). This indicates that the mitochondrial network structure of SCLC cells is more complex than normal cells. The increase of $D_{\left(Q\right)}$ on the left side and decrease of $D_{\left(Q\right)}$ on the right side of the SCLC sample after HGF treatment indicates the HGF treatment increases the mutli-fractal for SCLC samples. However, for normal cells, the HGF treatment reduces its multifractal property. Table 1 lists the generalized dimensions of the mutli-fractal analysis. The mitochondrial network of both normal cells and SCLC cells show a multifractal property. Table 1 shows the statistically significant differences between the normal cell and SCLC cell (without HGF treatment) for $D_{\left(0\right)}$ (*p =* 1.1 × 10^−5^), $D_{\left(0\right)}$ (*p =* 9.3 × 10^−7^), and $D_{\left(2\right)}$ (*p* = 6.9 × 10^−7^). The SCLC cell has a larger capacity dimension, which shows a more complex structure. The SCLC cell also has a larger information dimension, which shows a more uniform density. The larger correlation dimension of SCLC cells means its mitochondrial network has more compactness than normal cells. We observed the same pattern with HGF treatment. There are still the statistically significant differences between normal and SCLC cells for $D_{\left(0\right)}$ (*p =* 3.5 × 1^−6^), $D_{\left(1\right)}$ (*p =* 1.5 × 10^−6^), and $D_{\left(2\right)}$ (*p =* 9.8 × 10^−7^).

The singularity spectrum (*f*_(*α*)_*vs*.*α*) of normal and SCLC cells are shown in Figure 4C. Beside the capacity dimension difference we observed in the previous analysis, the singularity spectrum also shows that the width of the spectrum ($SW=\alpha_{max}-\alpha_{min}$) are different between SCLC and normal cells. The width of the spectrum is related to the heterogeneity of the local scaling index,  α [40]. In our mitochondria analysis, it provides information of the scaling diversity associated with the distribution of the mitochondrial network structure. Without HGF treatment, the normal cell (SW_BC_ = 0.9) has a wider range of spectrum than the SCLC cell (SW_DC_ = 0.87). After HGF treatment, the SCLC cell’s spectrum (SW_DH_ = 0.92) is larger than the normal cell (SW_BH_ = 0.88). Meanwhile, the HGF treatment also increases the SCLC cell’s spectrum range on both the left and right side. It indicates the increasing heterogeneity of the mitochondrial network in the SCLC cell after treatment. 

### 3.4. MitoMorF Can Discern Subtle Differences in Mitochondrial Dynamics

**Figure 5:**
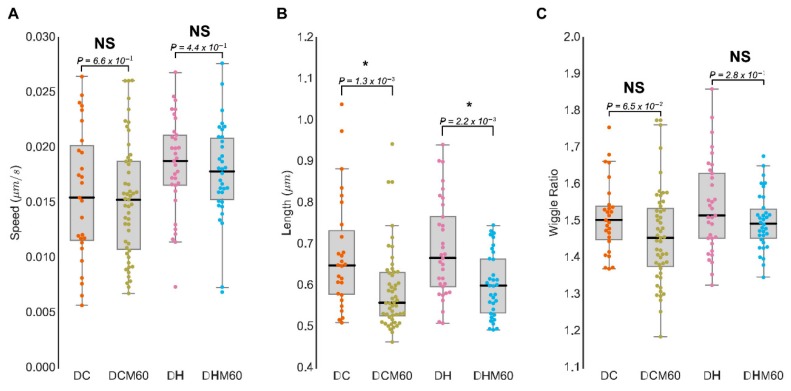
Mitochondrial network activity in live cells with and without MDIVI-1 treatment. The living cell mitochondrial video was captured using high resolution confocal laser scanning microscopy LSM880 and Airyscan-processed images were analyzed using Mytoe to measure speed (**A**), cell length (**B**), and wiggle ratio (**C**) after 60 min of 10 µM MDIVI-1 treatment. Box plots present the cell average of speed, length, and wiggle ratio. * *p* < 0.05 values indicate significance from the Mann–Whitney test in all three panels.

To evaluate the potential of MitoMorF to discern subtle changes in mitochondrial morphology and dynamics upon drug treatment, we also imaged the SCLC cells with or without drug treatment as described above. In total, 82 cells were evaluated following drug treatment. As shown in Figure 5, there were no discernable changes in mitochondrial speed, length, or wiggle ratios between the untreated and treated cells. However, mitochondrial length was significantly reduced after DRP1 inhibitor MDIVI-1 treatment in unstimulated DCM60 and HGF-stimulated DHM60 cell groups compared with untreated cells, suggesting that mitochondrial dynamics are important for regulation of function (Figure 5A–C). Furthermore, as shown in Table 2, multifractal analyses could tease out the small differences, although the changes were not statistically significant. However, multifractal analysis of the mitochondrial network after MDIVI-1 produced sigmoidal curves with behavior typical of multifractal structures (Supplementary Figure S3). As discussed earlier, if the image had monofractal status, the D_Q_ vs. Q spectra would be linear as Q increased and if it were not fractal in nature at all, the D_Q_ vs. Q spectra would have been a horizontal line.

### 3.5. Three-Dimensional Analysis of Mitochondrial Network to Identify Differences in Mitochondrial Morphology 

**Figure 6:**
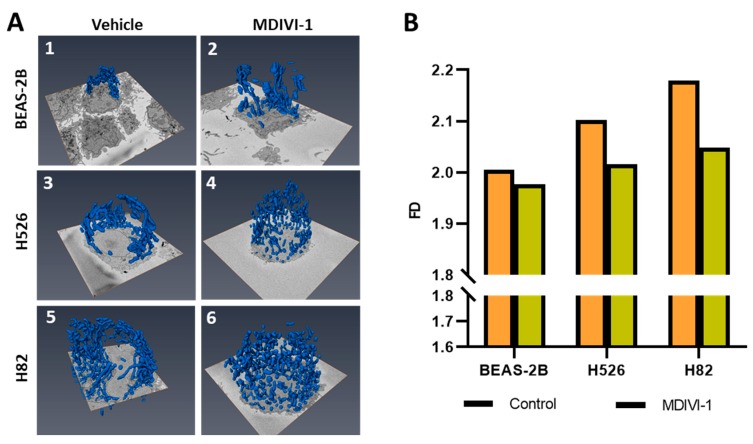
3-D reconstruction of mitochondrial network morphology with and without MDIVI-1 treatment: (**A**) We employed serial block-face scanning electron microscopy. We utilized three cell lines BEAS-2B normal lung epithelial and H82, H526 SCLC cell lines. The cell lines were observed under field-emission scanning electron microscope (Zeiss Sigma VP) with or without drug treatment (MDIVI-1 20 µM). An automatic microtome took 70-nm thick slices and this was repeated 1000 times to give a data set from which a complete three-dimensional reconstruction could be derived. Panels 1, 3, and 5 showed 3-D mitochondrial morphology in control BEAS-2B, H526, and H82, respectively. Patterns of the mitochondrial 3-D network in BEAS-2B and SCLC cells after MDIVI-1 treatment is shown on panels 2, 4, and 6. (**B**) Mono-fractal analysis of the 3-D reconstruction of the mitochondrial network in BEAS-2B, H526, and H82 cells.

Since the differences in mitochondrial morphology were indiscernible between the MDIVI1-treated and untreated BEAS-2B control cells with 2D confocal imaging analysis, we employed 3D reconstruction of SEM panels (Supplementary Figure S4) using normal epithelial BEAS-2B and two SCLC (H526 and H82) cell lines. As shown in Figure 6A, mitochondria in the untreated BEAS-2B cells (Figure 6A 1,2) appeared to be more tightly packed in a network while mitochondria in the MDIVI-1-treated cells looked more spread out in cellular milieu. However, there was no significant difference observed in control cells. On the other hand, mitochondrial morphology observed in SCLC cell lines (Figure 6A 3,4) had two major phenotypic characteristics. First the size and population of mitochondria in SCLC cells was larger than BEAS-2B control cells. Second, the cytoplasmic volume appeared to be markedly shrunk after MDIVI-1 treatment. The mitochondrial network appeared significantly disrupted and the individual mitochondria looked elongated. Fractal dimension measurement of the mitochondrial network showed the differences between BEAS-2B and SCLC cells (Figure 6B). The 3D fractal dimension analysis showed a 4.8% and 8.7% increase between BEAS-2B and H526 H-82, respectively. In the normal sample (BEAS-2B), the 3D FD only decreased 1.4% after MDIVI-1 treatment. In SCLC cancer samples, the 3D FD decreased an average of 5% after MDIVI-1 treatment. As we expected, BEAS-2B, with less mitochondrial network structure, had lower FD = 2.0047. In SCLC cell lines, we observed significant mitochondrial network changes in response to the treatment with MDIVI-1. MDIVI-1 treatment promoted mitochondrial fusion, resulting in reduced FD values in H526 and H82. 3D reconstruction of electron microscopy images and FD analysis demonstrated that SCLC cells had higher FD values than control BEAS-2B cells, consistent with the results obtained with the 2D model. 

## 4. Discussion

Understanding the processes of mitochondrial dynamics (fission/fusion) in live cells has been disadvantaged by the lack of automated methods to make measurements from microscopic images. In this manuscript, we present MitoMorF as a novel method to quantify mitochondrial morphology and dynamics employing high resolution confocal laser scanning microscopy and data analysis that combines video processing with Mytoe default filters to remove background noise. The method also captures the details of mitochondrial branch structures, and allows the measurement of mitochondrial size and the speed and patterns of movements. Moreover, the mitochondrial mask of the first frame of the video is used for fractal and multifractal analysis. Fiji/ImageJ’s Fraclac plugin [24] is used to calculate FD, lacunarity, and singularity spectrum. The gaps and the texture of the mitochondrial network images also provide important information about the heterogeneity of mitochondria in the cell. Lacunarity can be used to detect this image quantitatively. The larger and more irregular gaps are in the mitochondrial network, as observed in normal cells, and the greater is the value of lacunarity. Our data shows that FD plus lacunarity can be used as a discriminator of the mitochondrial network spatial distribution between normal and cancer cells. Further, as seen in the details of the statistics of the mean of generalized dimensions (Table 1), capacity dimension (D_0_), information dimension (D_1_), and correlation dimension (D_2_) showed a significant difference between non-cancerous and cancer cells. These results demonstrated that analysis of the FD of the cellular mitochondrial network is a reliable method to distinguish cancerous and non-cancer cells and hence, it is suitable for pharmacological and toxicological evaluation of novel drugs and diagnostics.

Although MitoMorF appears robust and looks promising, we do acknowledge potential limitations. For example, while the mitochondrial network parameters, namely speed and wiggle ratio, are statistically significant in some cases (Figure 3), they are insignificant in other experiments (Figure 5). One possible explanation is that while protein phosphorylation occurs rapidly, cytoskeletal rearrangement takes much longer, and the underlying complexities can be vastly different for these two events [41]. Furthermore, while protein phosphorylation could be governed by functional complexity, cytoskeletal rearrangement would be dictated by geometrical complexity. Further optimization of drug dose, imaging time interval, and frame rate could help eliminate such differences. Our method is based on 2D image analysis. Because the mitochondrial network in mammalian cells is not planar, 2D image analysis may not distinguish between tubules crossing in the z dimension and actual mitochondrial nodes. To reduce noise from the depth of the cell, during the living cell imaging capture, the Ziess LSM880 confocal microscope with Airyscan was configured with a 0.2AU (Airy Unit) pinhole size to reduce noise from the samples’ z dimensions. 

Of note, [42] proposed that each mitochondrial fusion event could be followed by a fission or secondary fusion event or that each fission could be followed by fusion or a secondary fission event. Consistently, measurement of fusion and fission events in neurons demonstrated that the fusion/fission are sequential events that form a cycle rather than independent, randomly occurring events. More importantly, these authors observed that the mean time interval of fusion/fission cycle was shorter (≈4.7 min) than the fission/fusion cycle (≈15.3 min) [42]. As our acquisition time was only ≈5 minutes, the longer cycle lengths could explain the lack of robust differences in fractal and mitochondrial parameters between untreated and drug-treated cancer cells in the present work. Increasing the acquisition time to 20 min with shorter intervals between frames, and measuring FDs, multifractal dimensions, and LC could distinguish the drug effects on mitochondrial morphology. Additionally, observing the entire mitochondrial network together with the changes in individual mitochondria may help discern subtle changes. Furthermore, as stated above, our 2D approach may be further improved and optimized by faster imaging and longer time points.

Therefore, we resorted to SEM to discern 3D images in order to reconstruct the mitochondria and hence, the network they form. This also allowed us to discern the effect of the drug on the mitochondrial network, which we could not do using the 2D approach. To the best of our knowledge, the application of fractal analysis on 3D-reconstructed images of mitochondria to determine the effect of subtle perturbations (such as the addition of a drug to the medium) on the mitochondrial network has not been reported. From the forgoing, it follows that our results demonstrate the feasibility of using mitochondrial fractal analysis as a readout of the physiological status of the cell. 

Notwithstanding the caveats mentioned here, we believe MitoMorF has significant translational potential. For example, imaging mitochondria in live circulating tumor cells (CTCs) obtained from SCLC patients that can be transiently cultured in vitro can provide valuable prognostic and predictive information to help clinicians make informed decisions regarding treatment options. Additionally, in other types of cancers where it is feasible to obtain patient-derived tumor samples, growing them as 3D spheroids or creating patient-derived xenografts (PDXs) in the zebrafish (zPDX), and subsequently imaging the cancer cells growing in the fish can also aid personalized treatment [43]. The zPDX option appears especially attractive when extremely small quantities of clinical samples are available, such as fine needle aspirates. 

## 5. Conclusions

We employed small cell lung cancer (SCLC) cells as a paradigm since they typically have very little cytoplasm but an abundance of smaller mitochondria compared to many of the commonly used cell types. We reasoned that if our method can be successfully applied to a challenging model like SCLC, it is more than likely to be successful with relatively fewer challenging ones. Indeed, our data demonstrate that mitochondrial morphology and dynamics can not only distinguish non-tumorigenic, control, and SCLC cells, but can also discriminate SCLC cells that are stimulated with human hepatocyte growth factor (HGF) c-MET agonist HGF from unstimulated SCLC cells. The rationale for testing the effect of HGF stimulation was that our previous studies on the SCLC cells established the multipurpose nature of MET/HGF pathway activation during tumor progression and invasion, which occurs via dysregulation of diverse biological functions, such as proliferation and differentiation, transcriptional control, cell-cycle G_1_/S checkpoint, cytoskeletal functions, survival, motility, and apoptosis [20]. In addition, we also utilized MitoMorF to discern subtle differences in SCLC mitochondrial dynamics in response to drug treatment. 

MitoMorF, a quantitative high-resolution confocal fluorescence microscopy imaging-based approach that combines automated mitochondrial morphology and dynamics with fractal analysis in live cells, is a powerful technique to discern subtle changes in mitochondrial dynamics. Such changes can serve as sensitive indicators of the cellular response to perturbations. The present study illuminates how knowledge derived from information-rich mitochondrial dynamics can be used to understand a cell’s adaptive response to the changing environment it inhabits. Further work in the future with more sensitive dyes, such as IraZolve-Mito, that can stain mitochondria in live or fixed cells, spheroids as well as fresh or fixed tissue samples, should help increase the resolution of the images and thus facilitate in-depth analysis using MitoMorF. These studies are currently underway in our laboratory. 

## Figures and Tables

**Table 1:** D_-10_, D_0_, D_1_, D_2_, and D_10_ of the mitochondrial network of BEAS-2B and SCLC cells in control and HGF-stimulated groups.

	Normal Cell	SCLC	*p*-Value (SCLC vs. Normal)	Normal Cell (HGF)	SCLC (HGF)	*p*-Value (HGF SCLC vs. HGF Normal)
D_-10_	2.077025	2.169330	4.8 × 10^−4^	2.049255	2.182030	4.7 × 10^−6^
D_0_	1.480179	1.584096	1.1 × 10^−5^	1.475005	1.573209	3.5 × 10^−6^
D_1_	1.423104	1.538348	9.3 × 10^−7^	1.419077	1.521315	1.5 × 10^−6^
D_2_	1.401221	1.519859	6.9 × 10^−7^	1.398245	1.499473	9.8 × 10^−7^
D_10_	1.346371	1.468496	3.2 × 10^−7^	1.343395	1.440852	3.2 × 10^−6^

Generalized dimensions of the mitochondrial network with *p*-values for SCLC vs. normal cells and HGF SCLC vs. HGF normal cells.

**Table 2:** D_-10_, D_0_, D_1_, D_2_, and D_10_ of the mitochondrial network of SCLC cells in control and HGF-stimulated groups before and after MDIVI-1 treatment.

	SCLC	SCLC with Drug	*p*-Value (SCLC with Drug vs. SCLC)	SCLC (HGF)	SCLC with Drug (HGF)	*p*-Value (HGF SCLC with Drug vs. HGF SCLC)
D_-10_	2.169330	2.151650	0.58	2.182030	2.129859	0.04
D_0_	1.584096	1.581537	0.60	1.573209	1.558646	0.22
D_1_	1.538348	1.526861	0.25	1.521315	1.505344	0.15
D_2_	1.519859	1.503207	0.15	1.499473	1.482131	0.10
D_10_	1.468496	1.446126	0.11	1.440852	1.422356	0.08

Generalized dimensions of the mitochondrial network with *p*-values for SCLC with drug vs. SCLC and HGF SCLC with drug vs. HGF SCLC.

## Supplementary Materials

The following are available online at https://www.mdpi.com/2077-0383/8/10/1723/s1, Figure S1: Representative images of immunohistochemical staining of MFN2 and BCL2 in human small cell lung cancer tissue microarray; Figure S2: Comparison of mitochondrial parameters in MDS273 cells before and after MDIVI-1 treatment in 60 min; Figure S3: Multifractal analysis of mitochondrial network after 60 min treatment with MDIVI-1; Figure S4: Electron microscopy; Table S1: SCLC Tissue Microarray IHC scores of DRP1 and stages of disease; Table S2: The values of mean Fractal Dimension (DB) and Lacunarity (Λ) of DRP1 stained control and small cell carcinoma lung tissue; Table S3: Scanning parameters for acquiring high resolution images of mitochondria; Video S1: BEAS-2B cells mitochondrial network was stained with MitoTracker and Hoechst; Video S2: BEAS-2B cells mitochondrial network was stained with MitoTracker and Hoechst; Video S3: DMS273 SCLC cells mitochondrial network was stained with MitoTracker and Hoechst; Video S4: DMS273 SCLC cells mitochondrial network was stained with MitoTracker and Hoechst; Video S5: DMS273 SCLC cells mitochondrial network was stained with MitoTracker and Hoechst; Video S6: DMS273 SCLC cells mitochondrial network was stained with MitoTracker and Hoechst.
