# Supplementary material for: Monitoring and Determining Mitochondrial Network Parameters in Live Lung Cancer Cells
Source: J Clin Med. 2019 Oct 18;8(10):1723. doi: 10.3390/jcm8101723 (PMC6832496; doi:10.3390/jcm8101723)
Supplement: Supplementary file 1 [file jcm-08-01723-s001.zip › JCM Manuscript supplemental data.pdf]

Supplemental Data

Supplemental Figure S1

A

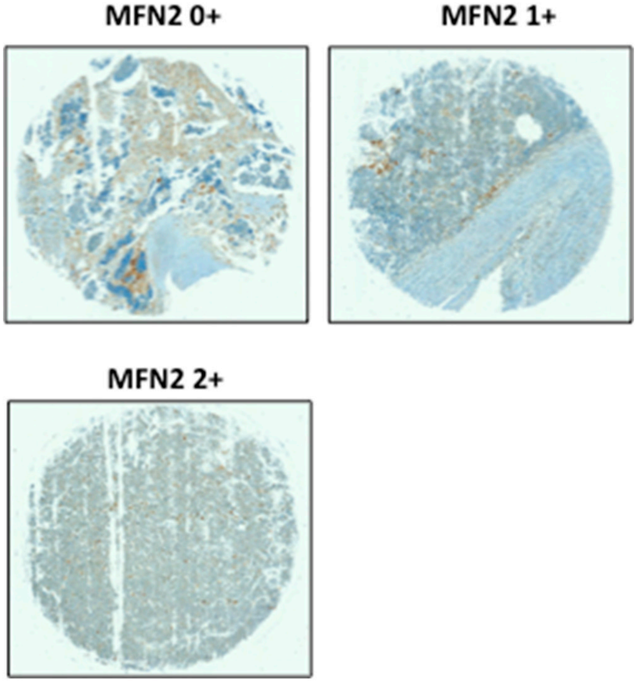

B

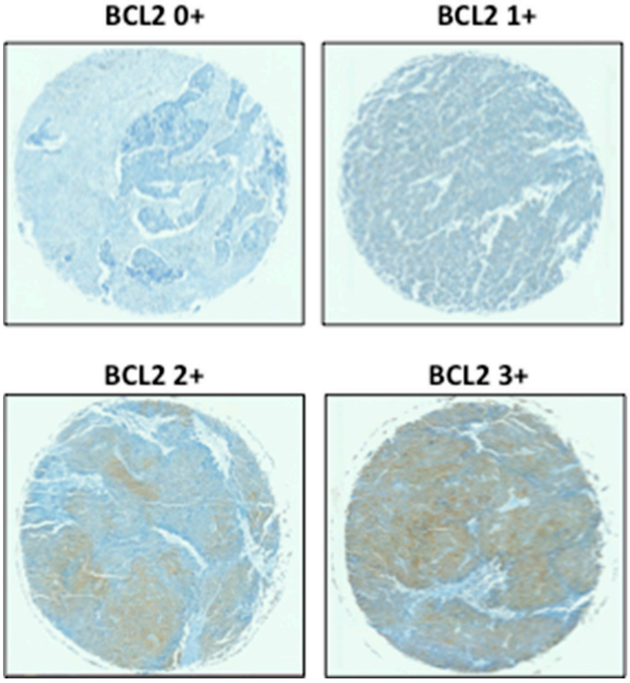

Supplemental Figure S1. **A and B.** Representative images of immunohistochemical staining of MFN2 and BCL2 in human small cell lung cancer tissue microarray. Detailed procedures are described in “Materials and methods.

Supplemental Figure S2

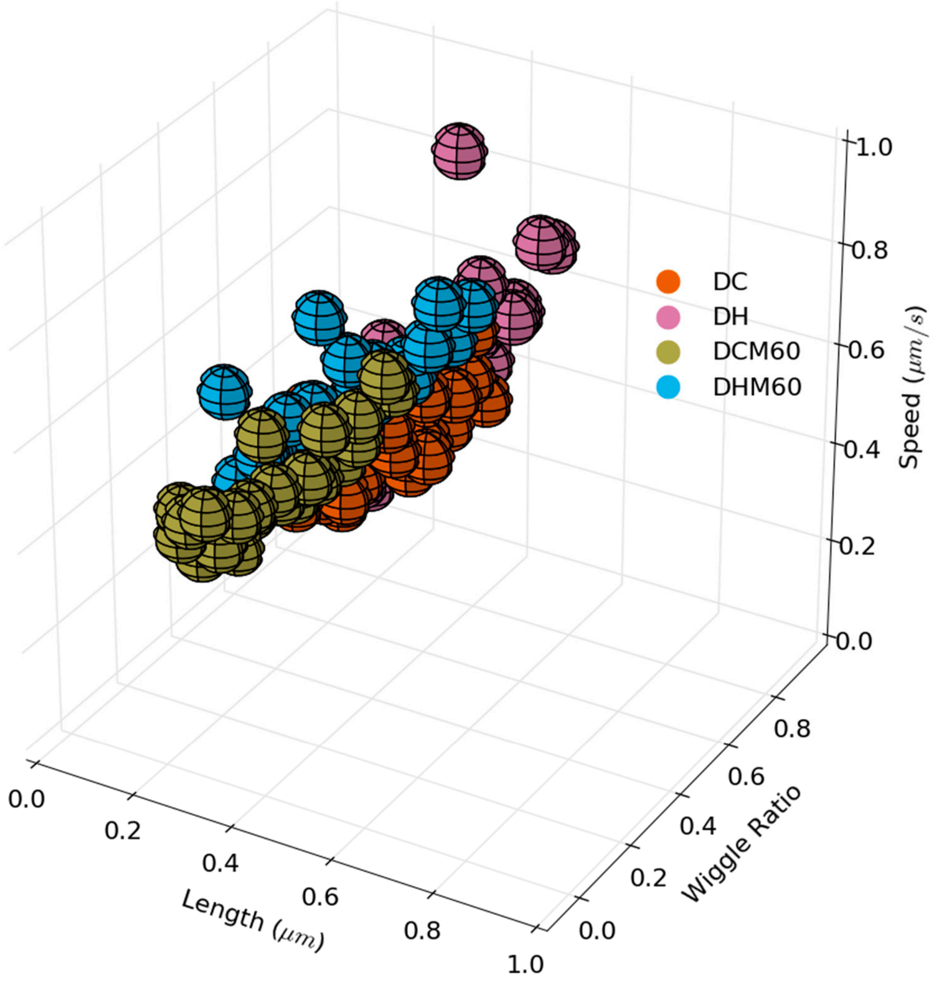

Supplemental Figure S2. Comparison of mitochondrial parameters in MDS273 cells before and after MDIVI-1 treatment in 60 min. 3D scatter plot shows the relationship between length (X-axis), wriggle ratio (Y-axis) and speed (Z-axis).

Supplemental Figure S3

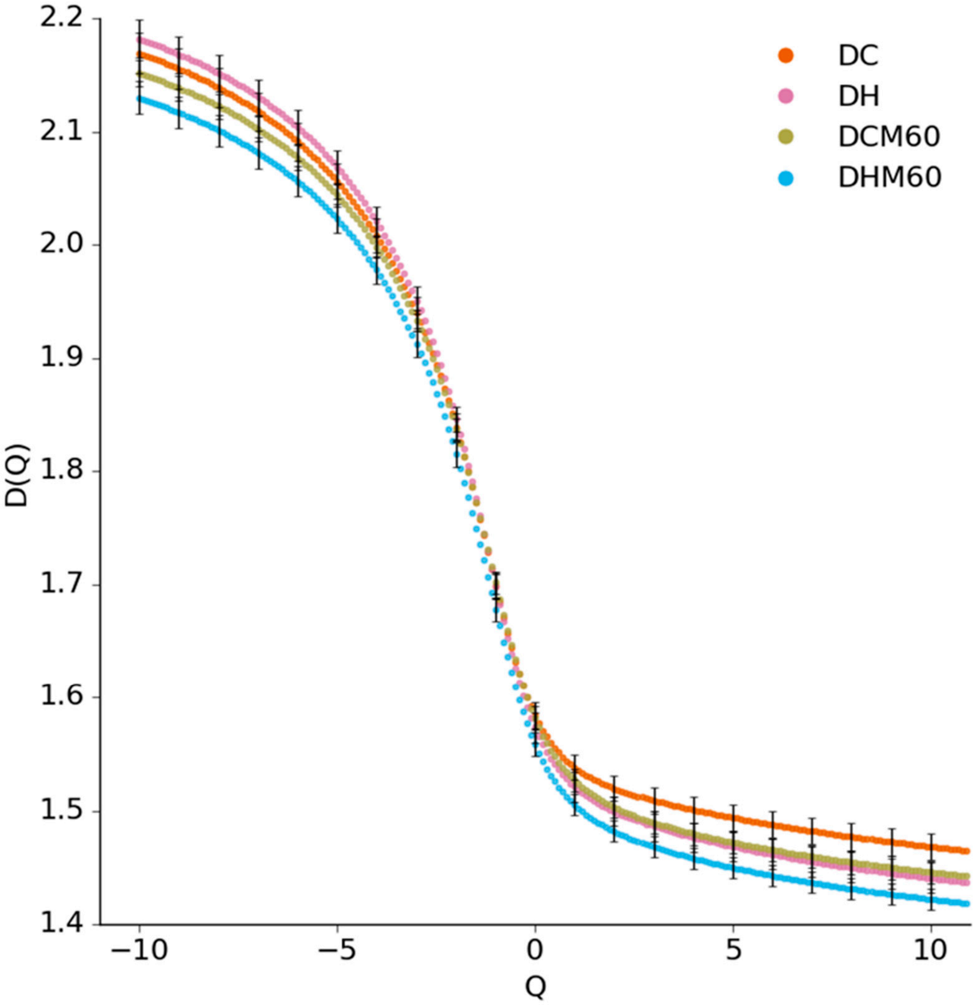

Supplemental Figure S3. Multifractal analysis of mitochondrial network after 60 min treatment with MDIVI-1. Generalized dimensions of all mitochondrial regions for BC-BH and DC-DH.

Supplemental Figure S4

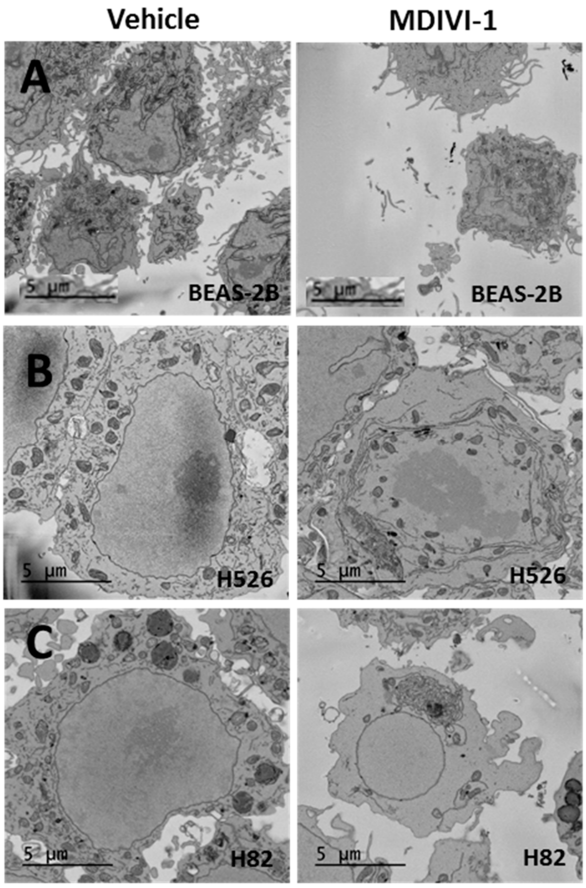

Supplemental Figure S4. Electron microscopy. Cells, cultured at 70% confluence, were fixed with 2.5% glutaraldehyde in 0.15 M cacodylate buffer pH 7.4. The cell pellet was then subjected to overnight infiltration in Dureapam Acm. Epoxy Resin (Electron Microscopy Sciences), flat embedding, and polymerisation at 55°C for 48 h. Embedded cells were mounted on resin stubs and sectioned at 70 nm with an Ultracut E ultramicrotome. Sections were viewed and photographed with a Zeiss Sigma VP. **A-C**: The pictures presented are representative for the experiment without and with MDIVI-1 (20 µM) treatment. 1000 images per cell was captured and used for three-dimensional reconstruction.

Supplemental Table S1. SCLC Tissue Microarray IHC scores of DRP1 and stages of disease.

Staining Intensity					
	**N**	**0+**	**1+**	**2+**	**3+**
**DRP1**	80	9 (Stage I)<br>1(Stage II)<br>0(Stage III)<br>0(Stage IV)<br><br>**10**	21(Stage I)<br>21(Stage II)<br>7(Stage III)<br>1(Stage IV)<br><br>**50**	9(Stage I)<br>6(Stage II)<br>4(Stage III)<br>0(Stage IV)<br><br>**19**	0(Stage I)<br>0(Stage II)<br>1(Stage III)<br>0(Stage IV)<br><br>**1**

Supplemental Table S2. The values of mean Fractal Dimension (D_B) and Lacunarity (Λ) of DRP1 stained control and small cell carcinoma lung tissue

**Sample**	**D_B**	**SE**	***P* value vs NL**	**Λ**	**SE**	***P* value vs NL**
NL	1.5392	0.0142		0.2861	0.0245	
I	1.6842	0.0020	<0.0001	0.1510	0.0246	<0.001
IA	1.6986	0.0058	<0.0001	0.1563	0.0105	<0.0001
IB	1.6963	0.0031	<0.0001	0.1487	0.0038	<0.0001
II	1.6926	0.0056	<0.0001	0.1680	0.0127	<0.0001
IIA	1.6764	0.0063	<0.0001	0.1500	0.0126	<0.0001
IIB	1.6824	0.0073	<0.0001	0.1718	0.0326	<0.0001
IIIA	1.6811	0.0089	<0.0001	0.1252	0.0094	<0.0001
IIIB	1.6942			0.0876		
IV	1.6849			0.1217		

IIIB and IV groups contain one tissue specimen per group.

Supplemental Table S3. Scanning parameters for acquiring high resolution images of mitochondria

Parameter	Value
Zoom	2
Pixel size	0.04 µm/pixel
Scan Speed	1.77 µsec/pixel
Pinhole	2 UA
Laser Intensity	561nm @ 1%, 405nm @ 5%
Detector gain	Red 800, Blue 857
