# Supplementary material for: Monitoring and Determining Mitochondrial Network Parameters in Live Lung Cancer Cells
Source: J Clin Med. 2019 Oct 18;8(10):1723. doi: 10.3390/jcm8101723 (PMC6832496; doi:10.3390/jcm8101723)
Supplement: Supplementary file 1 [file jcm-08-01723-s001.zip › JCM Movie legends.pdf]

Supplemental movie legends

Supplemental Movie 1
BEAS-2B cells mitochondrial network was stained with MitoTracker and Hoechst, as described in methods. Mitochondria were visualized using an inverted Carl Zeiss LSM880 utilizing the Airyscan Detector and an LCI-PlanNeofluar 63x/1.3 Immersion objective and Immersol (Carl Zeiss). The video is recorded on average 12 seconds per frame in order to capture super high resolution images (played at 20 fps). This movie shows mitochondrial network dynamic in control cells.

Supplemental Movie 2
BEAS-2B cells mitochondrial network was stained with MitoTracker and Hoechst, as described in methods. Mitochondria were visualized using an inverted Carl Zeiss LSM880 utilizing the Airyscan Detector and an LCI-PlanNeofluar 63x/1.3 Immersion objective and Immersol (Carl Zeiss). The video is recorded on average 12 seconds per frame in order to capture super high resolution images (played at 20 fps). This movie shows mitochondrial network dynamic in control cells with HGF stimulation.

Supplemental Movie 3
DMS273 SCLC cells mitochondrial network was stained with MitoTracker and Hoechst, as described in methods. Mitochondria were visualized using an inverted Carl Zeiss LSM880 utilizing the Airyscan Detector and an LCI-PlanNeofluar 63x/1.3 Immersion objective and Immersol (Carl Zeiss). The video is recorded on average 12 seconds per frame in order to capture super high resolution images (played at 20 fps). This movie shows mitochondrial network dynamic in DMS273 cells.

Supplemental Movie 4
DMS273 SCLC cells mitochondrial network was stained with MitoTracker and Hoechst, as described in methods. Mitochondria were visualized using an inverted Carl Zeiss LSM880 utilizing the Airyscan Detector and an LCI-PlanNeofluar 63x/1.3 Immersion objective and Immersol (Carl Zeiss). The video is recorded on average 12 seconds per frame in order to capture super high resolution images (played at 20 fps). This movie shows mitochondrial network dynamic in DMS273 cells with HGF stimulation.

Supplemental Movie 5
DMS273 SCLC cells mitochondrial network was stained with MitoTracker and Hoechst, as described in methods. Mitochondria were visualized using an inverted Carl Zeiss LSM880 utilizing the Airyscan Detector and an LCI-PlanNeofluar 63x/1.3 Immersion objective and Immersol (Carl Zeiss). The video is recorded on average 12 seconds per frame in order to capture super high resolution images (played at 20 fps). This movie shows mitochondrial network dynamic in DMS273 cells after one hour of MDIVI-1 treatment.

Supplemental Movie 6
DMS273 SCLC cells mitochondrial network was stained with MitoTracker and Hoechst, as described in methods. Mitochondria were visualized using an inverted Carl Zeiss LSM880 utilizing the Airyscan Detector and an LCI-PlanNeofluar 63x/1.3 Immersion objective and

Immersol (Carl Zeiss). The video is recorded on average 12 seconds per frame in order to capture super high resolution images (played at 20 fps). This movie shows mitochondrial network dynamic in DMS273 cells after one hour of MDIVI-1 treatment with HGF stimulation.
